# Challenges in establishing small animal models for *Ancylostoma caninum* : Host specificity and resistance to infection in rodent hosts

**DOI:** 10.17912/micropub.biology.001360

**Published:** 2024-12-24

**Authors:** Catherine A. Jackson, Elise L. McKean, John M. Hawdon

**Affiliations:** 1 Microbiology, Immunology, and Tropical Medicine, George Washington University, Washington, Washington, D.C., United States; 2 Biological Sciences, George Washington University, Washington, Washington, D.C., United States

## Abstract

This study explores potential small animal models for the dog hookworm, *Ancylostoma caninum* , a parasitic nematode which has repeatedly exhibited the ability to develop resistance to a range of anthelmintics. Immunomodulated hamsters, gerbils, rats, and mice were infected with *A. caninum.* Despite varying degrees of immunosuppression, and in some cases, total adaptive immunodeficiency, no adult worms were recovered, and larval arrest (L3 stage) occurred in muscle tissue of mice and hamsters. This highlights the strict host specificity of *A. caninum* and emphasizes the challenges of developing rodent models usable for anthelmintic testing with a strict specialist parasite.

**Figure 1. Corticosteroid administration schedules and infection outcomes for rodents infected with Ancylostoma caninum:**
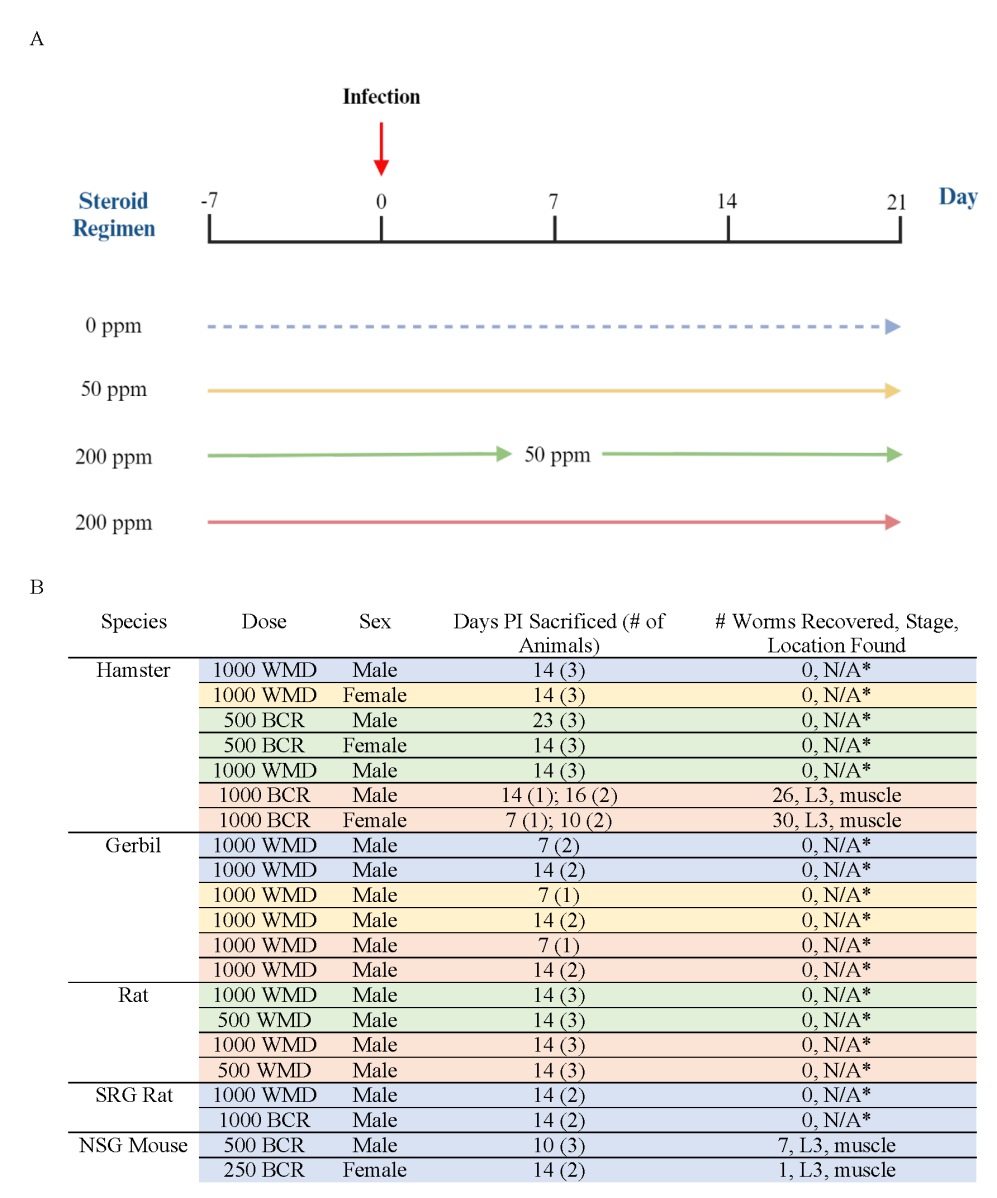
(A) Corticosteroid treatment schedules utilizing grain-based rodent feed milled with hydrocortisone acetate at concentrations of 50 and 200 parts per million. Administration of corticosteroid feed began 7 days before infection, day 0 being the day of infection. The four conditions include a high dose (red), an intermediate dose (green), a low dose (yellow), and a control (blue). (B) Infection outcomes for individual treatment groups within each of the five rodent species tested. The color of each row is consistent with the treatment schedules described in panel A. An asterisk is used to mark treatment groups in which only the small intestine was dissected.

## Description

The dog hookworm, *Ancylostoma caninum* , remains a significant pathogen of dogs, capable of causing severe anemia and even death in puppies and debilitated dogs (Bowman 2020) . This is exacerbated by the emergence and spread of naturally occurring multi-anthelmintic drug resistant (MADR) isolates (Kitchen et al. 2019; Jiminez Castro et al. 2019, 2020, 2021; Venkatesan et al. 2023; McKean et al. 2024) . The cost and ethical issues associated with anthelmintic testing in dogs and the strict host specificity of the parasite are serious impediments to the development of novel drugs to treat MADR hookworms. Development of a rodent model for *A. caninum* would obviate these obstacles. Success infecting immunodeficient mice with a generalist hookworm of the same genus, *Ancylostoma ceylanicum* , has been reported, despite mice with a fully functioning immune system not being a permissive host (Langeland et al. 2024) *.* Herein, we report results which indicate that the specificity of host-parasite interactions necessary for establishment in the host varies greatly among members of the same genus of nematode, as the specialist parasite *A. caninum* is unable to infect a non-canid host despite host immune suppression or deficiency.

Generation of small animal models for traditionally non-permissive hosts have been made possible through immunological modulation of a variety of rodent species. Such a model for *A. caninum* would greatly facilitate new drug development. Corticosteroid-treated Syrian hamsters ( *Mesocricetus auratus* ) can be successfully infected with the human hookworm *Necator americanus* (Sen and Seth 1970) , making them a promising target species for infection with *A. caninum* . Rats treated with corticosteroids support the development of several parasitic nematodes, including *Trichostrongylus colubriformis* (Gration et al. 1992) , *Strongyloides venezuelensis* (Gonçalves et al. 2010), and *Dirofilaria immitis* (Mills et al. 2020) . Gerbils ( *Meriones unguiculatus* ) effectively host the ruminant parasites *Trichostrongylus colubriformis* , *T. axei* (Leland 1963; Kates and Thompson 1967; Panitz et al. 1981; Ziam et al. 1999) , and *Haemonchus contortus* , with worm burdens increasing under immunosuppression (Gressler et al. 2019) . An immunosuppressed gerbil model has proven useful for testing anthelmintic efficacy, showing parallels in drug effectiveness between gerbils and sheep (Conder et al. 1990, 1991a, 1991b, 1992) . Gerbils are also susceptible to several non-natural nematodes, including *Strongyloides stercoralis* (Nolan et al. 1993) , S. *venezuelensis* (Couto et al. 2020) , *Teladorsagia circumcincta* (Court et al. 1988) , and *Nippostrongylus braziliensis* (Horii et al. 1993) . Given these documented susceptibilities to intestinal helminths, as well as the availability of immunodeficient rat and mouse strains, hamsters, gerbils, rats, and mice were tested as potential hosts for *A. caninum* under a variety of immunosuppressive conditions.

Syrian hamsters, a permissive host for *A. ceylanicum* , were treated with various regimens of hydrocortisone-enriched feed prior to infection and for the duration of the experiment (Figure). Following infection with both drug-resistant and susceptible *A. caninum* , no developed worms were recovered from the small intestine of any hamsters regardless of treatment group. A relatively small number of third stage larvae (L3) were found in the muscular tissue upon dissection. Given that no worms developed beyond the infective stage, it can be reasoned that a permissive host signal was absent and L3 migrated to the muscular tissue and arrested. While the developmental signal was absent in the hamster, the migration to the muscles suggests that hamsters can act as paratenic hosts.

Mongolian gerbils were treated with similar regimens of corticosteroid feed at various doses. Animals were infected with drug-susceptible *A. caninum* and the small intestine was dissected at either 7 or 14 days post-infection. Given that the prepatent period for *A. caninum* in a canid host is 14 days, this time point was selected for the determination of infection in the rodent hosts. Immunosuppression or genetic immunomodulation likely mitigates migration in the non-permissive host, and this timeline allows for the hypothesized development of L3 and increases the potential of seeing any developed worms before ejection from the host. Similarly to the hamsters, no developed worms were recovered in the small intestines of any gerbils. Muscle tissue was not dissected. Given the absence of adult worms in the intestines, it is likely that the L3 migrated to the muscle tissue, where they arrested development. This is a common survival strategy in paratenic hosts, in which the parasite is unable to complete its life cycle. In these hosts, *A. caninum* larvae enter a state of hypobiosis (developmental arrest) within the muscle tissue, potentially awaiting transmission to a definitive host by predation (Schad and Page 1982; Schad 1990) . Arresting behavior in the muscle tissue may allow the larvae to evade the immune response and remain viable for extended periods, facilitating their survival in unfavorable environments.

Separately from immunosuppression with corticosteroid feed, genetically immunodeficient rodents were also tested. Due to success with *A. ceylanicum* , NOD scid gamma (NSG) mice were considered a potential host for *A. caninum* . They are defective in several cytokine signaling pathways, have numerous flaws relating to innate immunity, and are devoid of mature T cells, B cells, and natural killer (NK) cells (Shultz et al. 1995, 2005, 2007) . There are fewer immunodeficient rat strains available, however an SRG *Rag2/Il2rg* double knockout exists that exhibits similar depletion of T, B, and NK cells (Hao and Rajewsky 2001; Song et al. 2010) . These immunodeficient lines of both rats and mice were infected with drug-resistant and susceptible *A. caninum* without administration of corticosteroid feed. Upon dissection of the small intestine at various intervals post-infection, no developed nematodes were observed. Dissection of the muscular tissue in NSG mice revealed a small number of arrested L3 consistent with hypobiosis in non-permissive paratenic hosts.

This study aimed to identify novel small rodent models to facilitate testing anthelmintics against MADR *A. caninum* . Despite the success of using immunodeficient mice as non-traditional hosts for *A. ceylanicum* , our results indicate that *A. caninum* is a strict specialist, unlike other members of the same genus. The failure of *A. caninum* to establish in any of the immunosuppressed or immunodeficient models tested indicates that the host immune system does not mediate host specificity in this hookworm and underscores the challenges in developing a reliable small animal model for this species. The strict host specificity limits the utility of traditional rodent models for life cycle maintenance and subsequent anthelmintic testing, which necessitates further exploration of novel approaches that can better mimic the canine host environment. Additionally, these results highlight how two closely related species have developed different survival strategies, and that the intricacies of the host-parasite relationship are far from understood.

## Methods

**Procurement and maintenance of *Ancylostoma caninum***

Isolates of both susceptible (WMD) and multi-anthelmintic drug resistant (BCR) *A. caninum* were maintained in beagles ( *Canis lupus familiaris)* infected with a sub-clinical dose of the hookworm. Feces containing hookworm eggs was collected from infected dogs, combined with an approximately 1:2 ratio of water and bone charcoal, and cultured in 150 x 15 mm petri dishes in an incubator at 27-30°C to allow the eggs to hatch and develop to infective L3s. At no earlier than 7 days after collection, coproculture plates were transferred to a Baermann funnel (Baermann 1917; Dinaburg 1942) filled with warm water. After approximately 24 hours, 50 mL of water from the funnel was collected. This liquid was washed twice with BU *bu* ffer (50 mM Na _2_ HPO _4_ /22 mM KH _2_ PO _4_ /70 mM NaCl) by centrifugation. After the final aspiration, the tube containing L3 was resuspended to 10 mL with sterile BU and stored in a 50 mL culture flask until used for infection **.**

**Corticosteroid-enriched feeding schedules**

Hydrocortisone acetate was milled into grain-based rodent diet at concentrations of 50 and 200 ppm. Aside from a control group fed standard rodent diet, three treatment schedules were utilized, from 7 days pre-infection to up to 23 days post-infection. The first schedule (low dose) consisted of 50 ppm feed for the duration of the experiment. The second schedule (intermediate dose) began with 200 ppm feed from 7 days before infection to 7 days after infection for a total of 14 days, then downshifted to 50 ppm feed for the remainder of the experiment. The third schedule (high dose) consisted of 200 ppm feed for the duration of the experiment.

**Infection of rodents**

On the day of infection, *A. caninum* L3 were surface sterilized using 1% (v/v) HCl in sterile BU for 30 minutes. After incubation, the nematodes were washed three times with Gibco PBS (phosphate buffered saline). Individual doses of 1000 L3 were administered to the animals by oral gavage. Rodents were infected with such an excess of iL3s based on the unlikely probability of *A. caninum* to establish in a non-canid host and create significant pathology. Animals were monitored daily for significant adverse effects for the length of the infection.

**Determining infection status**

At various times between 7- and 23-days post-infection, animals were sacrificed and dissected. The small intestine was excised from the abdomen, incised longitudinally along its entire length, placed in a large petri dish filled with PBS, and incubated at 37°C to facilitate the observation of any present nematodes. To determine if L3 were arresting in the muscles of the rodents, sections of muscle were removed and disassociated by cutting through the sections several times. The tissue was then placed in a mini-Baermann funnel overnight and incubated at 37°C (Huynh et al. 2022) . Following the 24-hour incubation, the tissue was discarded, and the liquid was centrifuged. Following aspiration of the supernatant, the remaining liquid was observed for larvae.

## Reagents

**Table:** 

Reagent	Details	Vendor
Hydrocortisone Acetate	CAS No. 50-03-4, 5g	Cayman Chemical (Ann Arbor, MI)
Corticosteroid feed, 50 ppm	F10214, Rodent Diet, Grain-Based (5V75), Hydrocortisone Acetate (50 mg/kg), Green, 1/2" Pellets, non-irradiated	Bio-Serv (Flemington, NJ)
Corticosteroid feed, 200 ppm	F10215, Rodent Diet, Grain-Based (5V75), Hydrocortisone Acetate (200 mg/kg), Red, 1/2" Pellets, non-irradiated	Bio-Serv (Flemington, NJ)
Bone Charcoal	50 lb bag 10 x 28	Ebonex Corporation (Melvindale, MI)
Gibco™ PBS	500 mL, pH 7.4 Catalog No. 10-010-023	FisherScientific (Boston, MA)

Animals:

**Table:** 

Species	Genotype/Strain	Vendor
Golden Syrian Hamster *(Mesocricetus auratus)*	HsdHan®:AURA	Envigo
Mongolian Gerbil *(Meriones unguiculatus)*	Crl:MON(Tum)	Charles River Laboratories
Sprague Dawley® Rat ( *Rattus rattus* )	Hsd:Sprague Dawley ^®^ SD ^®^	Inotiv
SRG Rat ( *Rattus rattus* )	Sprague Dawley- *Rag2 ^em2hera^ Il2rg ^em1hera^ /HblCrl *	Charles River Laboratories
NSG Mouse ( *Mus musculus* )	NOD.Cg- *Prkdc ^scid^ Il2rg ^tm1Wjl^* /SzJ	The Jackson Laboratory
